# Temporal distortion for angry faces: Testing visual attention and action preparation accounts

**DOI:** 10.1177/17470218231172856

**Published:** 2023-05-30

**Authors:** Jason Tipples, Michael Lupton, David George

**Affiliations:** 1 Psychology Group, Leeds Beckett University, Leeds, UK; 2 Department of Psychology, University of Hull, Hull, UK

**Keywords:** Temporal distortion, temporal bisection and emotion

## Abstract

When asked to judge the duration of a face people typically overestimate the duration of angry compared with neutral faces. A novel feature of the current research was the inclusion of secondary manipulations designed to distort timing performance namely the effects of visual cues (Experiment 1) and action preparedness (Experiment 2). Furthermore, to establish whether the effects are multiplicative with duration, the effects were examined across two duration ranges (200–800 and 400–1,600 ms). Visual cues and instructions to prepare to act increased the tendency to judge faces as lasting longer. Experiment 1 revealed an unexpected underestimation effect for angry faces presented for short durations (200–800 ms). However, the effect was not replicated in Experiment 2 where the results were generally consistent with either an increase the speed of a pacemaker mechanism that resides within an internal clock or the widening of an attentional gate—the temporal overestimation effect for angry faces grew in magnitude from the short to long duration. Experiment 2 also showed that the temporal overestimation for angry faces was reduced in magnitude when participants were asked to prepare to either push or pull a joystick.

Research indicates that our perception of time can be distorted for emotionally arousing events (e.g., Angrilli et al., 1997; Bar-Haim et al., 2010; Gil & Droit-Volet, 2011; Tipples, 2011). Such effects have been interpreted within the framework of scalar expectancy theory (SET; Church & Deluty, 1977; Gibbon, 1977; Gibbon et al., 1984). SET specifies an internal clock comprised of (1) a pacemaker that emits units of time (or pulses) at a variable rate, (2) an attention-controlled switch that closes when timing starts and opens when timing ends, and (3) an accumulator where perceived time is calculated based on the total number of counted units. Within the internal clock specified in SET, once counted units of time enter the accumulator, they are compared with a reference duration that is held in working memory.

A specific prediction of SET is that when pacemaker speed increases (e.g., due to emotional arousal) then the number accumulated pulses will also increase leading to a multiplicative relationship within duration. Closure of the switch is thought to occur once, before timing starts, and consequently, delays in the closure of the switch are expected to be additive with increases in time. In the later attentional gate model of prospective timing (Zakay & Block, 1996), an attentional gate precedes the switch. The gate can be opened at variable widths, depending on the importance and relevance of time. When timing is important, the gate is opened wider, and consequently, the number accumulated pulses increase as duration lengthens. This provides a second way in which emotion might produce a multiplicative effect namely via a widening of the attention gate. Overall, an additive pattern for emotion supports an attentional switch whereas a multiplicative pattern could be due to either (1) a widening of the attentional gate or (2) speeding of a pacemaker mechanism or (3) both widening of the gate and pacemaker speeding.

Studies using the temporal bisection task have reported mixed evidence for a multiplicative effect across duration ranges (Droit-Volet et al., 2010, 2011; Fayolle et al., 2015; Grommet et al., 2011, 2019; Smith et al., 2011). One study (Gil et al., 2007) in which children aged 3, 5, and 8 were asked to judge the duration of angry and neutral facial expressions, and recorded an additive pattern. However, multiplicative effects have been recorded for neutral stimuli following film-induced mood (Droit-Volet et al., 2011) and when participants are anticipating an electric shock (Droit-Volet et al., 2010). For studies in which participants judge the duration of static images (e.g., the pictures of threatening animals), some studies have reported a multiplicative effect (Grommet et al., 2019) and other studies have recorded an additive effect (Grommet et al., 2011).

One study (Smith et al., 2011) reported an underestimation effect for images presented for very brief durations (100–300 ms). The authors argued that such brief duration effects coincide with the influence of emotional images on attention within the extrastriata cortex (Gan et al., 2009). Furthermore, the authors speculate that such modulatory effects likely originate in the amygdala where the emotional stimulus is immediately identified as a threat. Subsequently, the amygdala modulates other brain systems.

## Secondary manipulations

A further way to strengthen the conclusion that either switch or pacemaker is responsible for the effects of emotion on time is to include a separate manipulation that targets a specific component of the internal clock. For example, a manipulation such as the presentation of a visual cue before the to-be-timed stimulus should reduce switch closure latency leading to an additive effect across duration ranges. If emotion speeds a pacemaker or widens an attentional gate, then the two effects will be separable—there will be a main effect for the manipulation of attention and a duration range × emotion interaction (the latter reflecting a speeding of the pacemaker). Alternatively, if emotion speeds switch closure latency, then facilitating switch closure by increasing attention should effectively reduce or eliminate the differences between the emotion conditions—a cue × duration × emotion interaction reflecting a reduction in the effect of emotion in the cue conditions. Other relevant manipulations include preparing to act, a manipulation that produces similar overestimation effects to the effects of emotional arousal (Hagura et al., 2012; Iwasaki et al., 2017).

## Overview

This study filled a gap in the literature by (1) testing for a multiplicative effect of emotion across duration ranges using angry and neutral facial expressions and (2) concurrently validating other aspects of the internal clock through secondary manipulations of visual attention (Experiment 1) and action preparation (Experiment 2). Participants in this study judged the duration of angry and neutral expressions displayed for durations ranging from either 200–800 or 400–1,600 ms. Including the duration range 400–1,600 ms matters because this is the duration range at which studies have consistently reported an overestimated effect for angry compared with neutral expressions. Therefore, the study includes a condition that permits the replication of past findings but also allows for the testing of the clock-speeding hypothesis.

In Experiment 1, visual attention was manipulated using three trial types: (1) no-cue, (2) central visual cue, and (3) peripheral visual cue. On central and peripheral cue trials, a brief (200 ms) flickering visual cue was displayed before the to-be-timed face. By comparing the effects of peripherally presented cues with centrally presented cues and a no-cue condition, we aimed to establish whether the effects of cues are due to either spatially selective or nonspatially selective attention. If the effects require spatially selective attention, then presenting a peripheral cue away from fixation should delay attention to the face, and consequently, timing will start later—there will be a delay in switch closure required before timing can start. In terms of timing performance, if a peripheral cue attracts attention away from the to-be-timed faces, then the prediction is that time will be underestimated (fewer long responses) for the peripheral cue compared with the central and no-cue conditions. Alternatively, the effects of cues might be nonspatially selective reflecting a general increase in alertness across the visual field (Fernandez-Duque & Posner, 1997). If the effects lead to an increase in general (nonspatial) alertness, then the prediction is that both the central and peripheral cue conditions will either speed the pacemaker (or reduce switch closure latency) leading to an overestimation of time compared with the no-cue condition.

### Indices of timing and SET

We calculated standard indices of timing performance for the bisection task namely the bisection point (BP), just noticeable difference (JND), and Weber ratio (WR). These were derived from the intercept and slope of the psychometric curves for each condition. The WR is a standardised index of temporal sensitivity. The WR can be calculated by dividing the difference limen [(p(long) = .75 − p(long) = .25)/2] by the BP. JNDs were calculated using the following equation: log(0.75/(1–0.75))/slope, where “slope” is the estimated psychometric curve calculated from a logistic or probit regression model. The current research includes predictions for equivalence. Here, we tested for equivalence using two methods namely (1) graphical superimposition and (2) comparison of Bayes factors. Superimposition is tested for by normalising the psychometric functions by the correspondent BP for each duration range. Superimposition implies the scalar property whereby the standard deviation (*SD*) of the mean of estimations for different durations varies as a constant fraction of their mean (Gibbon & Church, 1990)—the slopes for each duration range should be equivalently steep (they should superimpose) once duration has been normalised.

In addition to graphical superimposition, we calculated Bayes factors so that we could make quantitative statements about the extent to which two models are equivalent (for alternative Bayesian approach see; Lee & Wagenmakers, 2013; Tipples, 2019). For example, if SET holds, then WRs are expected to be equivalent across duration ranges (Bayes factor analyses will favour the null model without duration range)—whereas JNDs are expected to be larger for the long-duration range (Bayes factor analyses will favour the model with duration range as a variable).

### Method

#### Participants

A total of 56 undergraduate psychology students (33 females, mean age = 21.33, *SD* *=* 1.89; 23 males, mean age = 21.21, *SD* *=* 2.86) from the University of Hull participated in return for a course credit. All had normal or corrected to normal vision, and 22 were right-handed. Prior to commencing the experiment, ethical approval was granted from the Psychology Departmental Ethics Committee.

#### Stimuli and apparatus

A total of 16 digitised photographs from the NimStim set of facial expressions (Tottenham et al., 2009) of four males and four females each displaying an angry and neutral facial expression were used. When presented in the centre of the computer screen, at an average distance of 60 cm, the faces measured 18° of vertical visual angle. An identical flickering cue was presented on both noncentral and central cue trials. The flickering cue was a white oval and subtended 18° of vertical visual angle and 11° of horizontal visual angle and flickered at 50 Hz for 200 ms (a 10 ms white display followed by a 10 ms blank display repeated 10 times). On noncentral cue trials, the horizontally displaced cue was shifted 10° of horizontal visual angle to the right and left did not overlap with the to-be-time face stimuli.

All stimuli were presented on a 17-inch computer monitor (1,280 × 1,080, 60 Hz) connected to a 1 GHz Pentium computer. Stimulus presentation and data collection were controlled by E-Prime software (Schneider et al., 2002).

#### Procedure

All participants completed a learning and test phase. On entering the laboratory, participants were randomly assigned to one of two duration ranges (short range: 200, 300, 400, 500, 600, 700, and 800 ms; long range: 400, 600, 800, 1,000, 1,200, 1,400, and 1,600 ms). In the learning phase, participants were trained to discriminate short (either 200 or 400 ms) from long (either 800 or 1,600 ms) stimulus durations. On the first eight trials, a pink oval appeared for either a short or long duration in a fixed sequence (e.g., long–short–long–short). Participants were told to expect this sequence and to press either the z or m to indicate whether the oval appeared for either a short or long duration. The response mapping (e.g., z for short durations and m for long durations) was counterbalanced across participants. Following a response, participants were presented with visual feedback lasting 500 ms, for both correct (“yes”) and incorrect (“no”) decisions. The feedback was followed by a fixed 1,000 ms intertrial interval. In the final stage of the learning phase, the pink oval was presented for a further eight trials in a new random order for each participant. Participants continued to indicate whether the oval appeared for either short or long stimulus durations and received feedback.

During the test phase, the oval was replaced by the face stimuli. Participants were asked to (1) look at the face and (2) indicate whether the face appeared for a duration that was closer to either the short or long durations that they had learned earlier. Feedback was not given during the main test phase. In the test phase, there were 42 possible trial types that were derived from the factorial combination of duration (7) × expression (2; angry and neutral) × cue condition (3; central, noncentral, and no-cue). Each of the eight neutral and eight angry expressions were displayed for the standard short and long durations, and a range of intermediate durations (short range: 300, 400, 500, 600, and 700; long range: 600, 800, 1,000, 1,200, and 1,400) in both the cue and no-cue conditions leading to the creation of 336 trials. On noncentral cue trials, the direction of horizontal displacement (left or right) was determined on each trial according to a uniform random distribution. A new randomised trial order was created for each participant.

### Results

To estimate a psychometric curve for each person for each combination of expression, cue, and duration range, the [*p(*long)] responses were modelled using a binomial generalised linear model (GLM) with a logistic link function.

#### Model selection and *t*-tests

Model comparison for BPs, JNDs, and WRs was conducted by calculating Bayes factors. Bayes factors were calculated using the BayesFactor package in R (Morey & Rouder, 2018) and more specifically, by using the default settings of the generalTestBF function to compare every model against a general-intercept-only model. Models included all possible combinations of cue type, expression, and range and also, by-participants effects that are normally included as residual/error terms in frequentist analysis of variance (ANOVA) (van Doorn et al., 2023). Bayes factors were calculated using the Jeffreys–Zellner–Siow priors: a noninformative Jeffreys prior on the variance of the population and a Cauchy prior with default scales on the standardised effect size for effects (Rouder et al., 2012). Following Jeffery’s scheme Bayes factors (BF10) for the alternative hypothesis were classified as either “anecdotal” (1–3), “moderate” (3–10), “strong” (10–30), “very strong” (30–100), or “decisive” (>100). With respect to the null hypothesis, Bayes factors (BF10) were classified as either “anecdotal” (1–0.33), “moderate” (0.33–0.1), “strong” (0.1–0.03), “very strong” (0.03–0.01), or “decisive” (<0.01). Differences were considered “significant” or noteworthy if BF10 values offered at least “anecdotal” support for either the null or alternative hypothesis.

Tests of differences were conducted using a one-sample Bayesian *t*-tests to estimate both a standardised effect size Cohen’s *d*_z (_µ − 0/σ) and the 95% highest density interval (HDI) around the effect size difference from zero. The BEST package (Kruschke & Meredith, 2021) was used with for the latter calculations. The Bayesian Estimation Supersedes the t-Test (BEST) uses minimally informative priors with normal priors with large *SD* for µ, broad uniform priors for σ, and a shifted-exponential prior for ν. More details can be found in Kruschke’s study (2018). Cohen (1988) provided guidelines for interpreting the magnitude of effect sizes namely Cohen’s *d*_z_ = 0.2 (small), Cohen’s *d*_z_ = 0.5 (medium), and Cohen’s *d*_z_ = 0.8 (large). A region of practical equivalence (ROPE) was defined as effect sizes falling within Cohen’s *d*_z_ _=_ −0.1 to 0.1. A 95% HDI that falls outside this region might be considered “statistically significant.”

**Figure 1.**
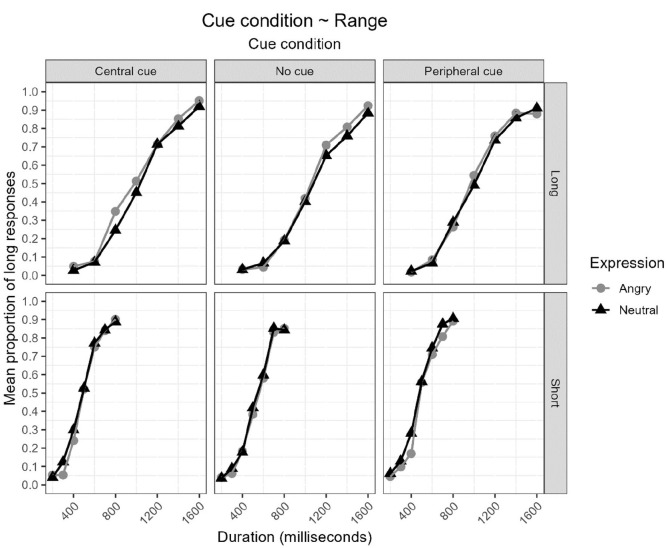
Proportion of long responses [*p*(long)] plotted against durations (ms) for the short-duration range group (bottom) and long-duration range group (top) for each combination of cue condition (central, no-cue, and peripheral cue) and expression (angry and neutral).

Figure 1 displays the proportion of long responses [*p*(long)] plotted against durations (ms) for the short-duration range group (top) and long-duration range group (bottom) for each combination of cue condition (central, no-cue, and peripheral cue) and expression (angry and neutral).

##### BPs

The four models with the most support relative to the null are shown in Table 1 (top). Bayes factor analyses determined that the data were best represented by a model that included the main effects of expression, (duration) range, cue, and the expression × range interaction term. The Bayes factor (BF10) was 1.44 × 10^168^ indicating decisive evidence in favour of this model when compared with the null model. Evidence favouring the top model was “moderate” when compared with the second model as shown in Table 1. The second model included the additional expression × cue interaction term.

**Table 1.** Bayes factors relative to null (grand mean and intercept only) for BPs, WRs, and JNDs for Experiment 1. The four models with the most support for each index (e.g., BPs) relative to the null are presented in rank order.

Model comparison	
BP models	BF10
1. Expression + Cue + Range + Expression:Range + Cue:Subject + Subject	1.44 × 10^168^
2. Expression + Cue + Expression:Cue + Range + Expression:Range + Cue:Subject + Subject	3.24 × 10^167^
3. Expression + Cue + Range + Expression:Range + Cue:Range + Cue:Subject + Subject	2.88 × 10^167^
4. Expression + Cue + Range + Expression:Range + Subject	7.65 × 10^166^
WR models	
1. Subject	5.81 × 10^30^
2. Range + Subject	3.15 × 10^30^
3. Expression + Subject	2.33 × 10^30^
4. Cue + Subject	2.81 × 10^30^
JND models	
1. Range + Subject	8.41 × 10^61^
2. Expression + Range + Subject	2.43 × 10^61^
3. Expression + Subject	5.03 × 10^60^
4. Cue + Subject	4.25 × 10^60^

BP: Bisection point; JND: just noticeable difference; WR: Weber ratio.

**Figure 2.**
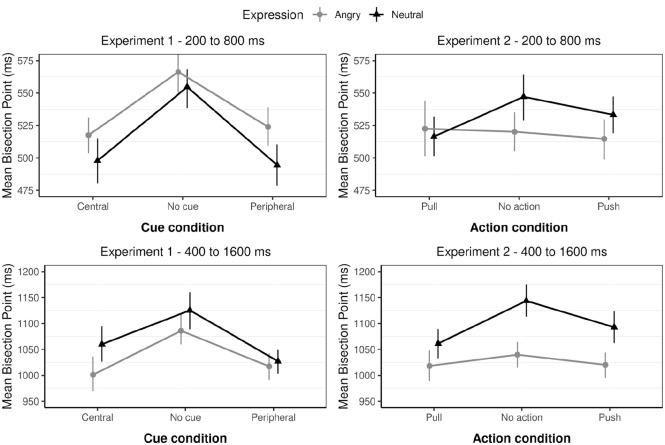
The mean BPs in ms for Experiments 1 (left) and 2 (right) for the short-duration range (top row) and long-duration range (bottom row) conditions as a function of expression (angry and neutral) and either cue condition (Experiment 1) or action condition (Experiment 2). Error bars are bootstrapped standard errors.

As shown in Figure 2 (left column), the expression × range interaction showed that for the short duration, the BP was reached later for angry (*M* *=* 535) compared with neutral (*M* *=* 515) expressions, Cohen’s *d*_z_ = 0.67, 95% HDI = [0.23, 1.14], whereas for the long-duration range, the BP was reached sooner for angry (*M* *=* 1,034) compared with neutral (*M* *=* 1,070) expressions, Cohen’s *d*_z_ = −0.66, 95% HDI = [−1.14, −0.20]. In both cases, less than 1% of the HDI for the effect sizes fell within the ROPE (Cohen’s *d*_z_ _=_ −0.1 to 0.1).

Also, as shown in Figure 2, BPs were much lower in the short duration (*M* *=* 525) compared with the long duration (*M* *=* 1,052), Cohen’s *d*_z_ = −4.04, 95% HDI = [−4.55, −3.55]. The effect of cue condition showed that BPs were reached sooner for both the central cue (*M* *=* 769) and the peripheral cue (*M* *=* 765) compared with the no-cue condition (*M* *=* 833). Specifically, Bayesian *t*-tests indicated a medium-to-large, standardised effect sizes for both the central cue versus no-cue contrast (Cohen’s *d*_z_ = −0.74, 95% HDI = [−1.07, −0.44]) and the peripheral versus no-cue contrast (Cohen’s *d*_z_ = −0.74, 95% HDI = [−1.08, −0.43]). A Bayesian *t*-test comparing the central cue versus peripheral cue condition revealed a very small effect (Cohen’s *d*_z_ = −0.06, 95% HDI = [−0.35, 0.217]) with 32% of the effect falling with the ROPE—the BP difference was practically equivalent to zero.

**Figure 3.**
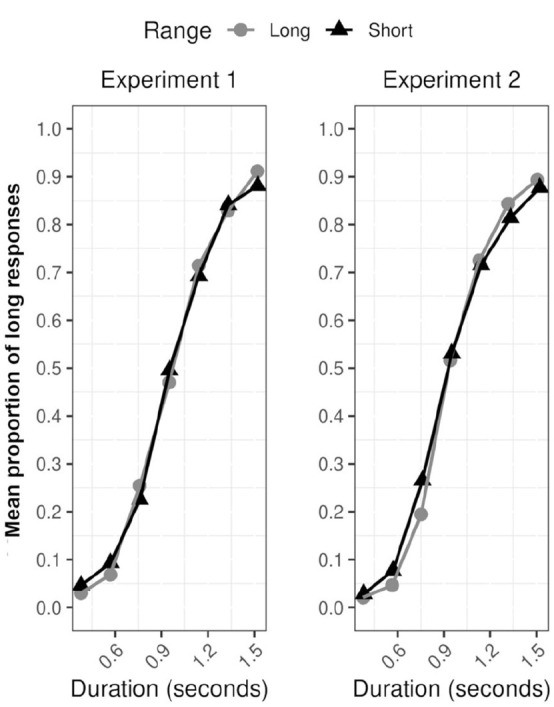
Superimposition—proportion of “long” responses, plotted against stimulus duration divided by the appropriate BP for each duration range and each experiment separately.

SET predicts changes in the absolute but not standardised indices of temporal sensitivity across duration ranges. Therefore, absolute differences in temporal sensitivity are predicted for JNDs (JNDs should be larger for the long-duration range), and moreover, the proportion of long responses for each duration range plotted against the normalised index for duration (the duration divided by the BP) should superimpose. As shown in Figure 3 (left), the data appear to show superimposition supporting scalar invariance (but see below).

As a more formal statistical test for superimposition, the proportion of long responses for each range and transformed duration (0.3, 0.5, 0.7, 0.9, 1.1, 1.3, and 1.5) were modelled in Bayesian multilevel logistic regression. The model included the default weakly regularising priors included in the rstanarm package for *R* (Goodrich et al., 2020). The model included by-participants varying intercepts and by-participants varying slopes for the transformed duration variable. A correlation between the by-participant varying intercepts and slopes was also included in the model. In the model, the group-varying (fixed) effects were *b*-transformed duration, *b* range (short) and the *b*-transformed duration × range (short) interaction were regressed onto the proportion of long responses. The critical test of the scalar invariance assumption is the *b*-transformed duration × range (short) interaction term.

For the interaction term, the 95% HDI included the value zero, *b*-transformed duration × range (short) Log Odds *=* −0.51, [−1.35, 0.30]—as might be expected the gradient for duration remains constant across duration ranges. To supplement the analyses, the probability of direction (pd) (Makowski et al., 2019) was also calculated for the interaction effect. The pd is a measure of effect existence representing the certainty with which an effect is positive or negative. The pd for the interaction term was 89.4%. In other words, a high proportion of the posterior distribution was negative—we can be 89% certain that the effect is negative representing a flatter slope in the short-range condition.

JNDs and WRs were subjected to the same Bayesian factor analyses used for the BPs. Bayes factor analyses are shown in Table 1. Bayes factors favoured the model that include only the by-participants intercepts (without duration range), BF10 = 5.81 × 10^30^ against the model with the grand mean only (Table 1—middle row). However, the model with the second highest BF did include the variable duration range, BF10 = 3.15 × 10^30^. For the JNDs, the best-fitting model included duration range as the sole factor. Specifically, JNDs were smaller for short-duration group (*M* = 112) compared with the long-duration range group (*M* = 199 ms), and the difference was large, Cohen’s *d*_z_ = −1.810, 95% HDI = [−2.77, −0.93]. For WRs, the evidence favouring best-fitting model versus the second, third, and fourth best-fitting models was anecdotal (BF10 >1 and BF10 <3). Furthermore, model comparisons for JNDs (see Table 1, bottom row) showed that evidence favoured the model with duration range as the sole factor relative to the second, third, and fourth best-fitting models (Table 1) with evidence ranging from moderate to strong.

### Discussion

Visual cues produced an additive overestimation effect across duration ranges a finding that is consistent with a mechanism responsible for increasing attention to the to-be-timed stimulus, and consequently timing starting sooner. For the effect of expression, timing performance in the long-duration range (400–1,600 ms) condition replicated the pattern found in numerous studies namely an overestimation effect relative to neutral cues—BPs were reached sooner for angry versus neutral expressions. Unexpectedly, the effect was reversed in the short-duration range (200–800 ms) condition—angry expressions were underestimated relative to neutral expressions when presented for brief durations. The underestimation effect deserves replication. In Experiment 2, we sought to replicate the underestimation effect for angry relative to neutral faces using different faces. Specifically, in Experiment 2, we used computer-generated faces (Tipples, 2007) shown in previous research (Tipples, 2011) to lead to a large, reliable overestimation effect relative to neutral facial expressions.

## Experiment 2

Following Experiment 1, Experiment 2 also included a secondary manipulation. Specifically, we wanted to establish whether emotion cues might tap later motor processes rather than early visual orienting and, therefore, included manipulation of “action preparation.” Research indicates that preparation to act leads to similar overestimation effects as that reported for emotion (Hagura et al., 2012; Iwasaki et al., 2017). This similarity may be more than superficial. For example, emotional arousal due to seeing a threatening face might prepare the observer to “fight or take “flight.” If emotion and preparation to act share a common process, then we might expect the two variables to interact with, for example, instructions to prepare to act either reducing or increasing the effects of emotion. To manipulate action preparation, on some trials participants we required to either prepare to act (by pushing or pulling a joystick) or not act by “holding still.” Compared with the no-action condition, the BPs should be reached sooner when participants are asked to either prepare to pull or push the joystick—preparing to act should lead to relative overestimation as reported elsewhere (Hagura et al., 2012). If emotion and action preparation share a common process, then the differences between emotion conditions should reduce in magnitude when participants are instructed to pull or push the joystick.

A more complex variant of this hypothesis is that participants will consider pulling and pushing the joystick as emotionally meaningful—the direction of the action may have motivational relevance. For example, as suggested by a separate line of research (Marsh et al., 2005; Rotteveel & Phaf, 2004) in which participants were faster to pull a level in response to happy expressions and push a lever in response to angry expressions, the act of pushing might reflect avoidance whereas the act of pulling might reflect approach. Pulling and pushing may lead to differential effects on time estimates too with, for example, underestimation for puling and overestimation for pushing. Our experiment allows for the exploration of such differences with differences in the direction of temporal overestimation effects occurring between the push and pull conditions. We do not have specific hypotheses for such interaction effects.

### Method

A total of 76 participants took part in the experiment. Participants were randomly assigned to take part in either the short-range condition (37 females, mean age = 21.48; 4 males, mean age = 21.21) or the long-range condition (23 females, mean age = 19.43; 12 males, mean age = 22.25). All participants were psychology undergraduate students attending the University of Hull. Participants received course credit for taking part in the experiment. Prior to commencing the experiment, ethical approval was granted from the Psychology Departmental Ethics Committee.

#### Stimuli and apparatus

The facial stimuli used had been created by commercial company DAZ Productions, Inc., Draper UT for use with the software program Poser 5.0 (Curious Labs Inc., Santa Cruz, CA). The facial stimuli were modified to show a threatening and neutral expression and are shown in the Supplemental Materials. The threatening facial stimulus has been used in previous research (e.g., see Tipples, 2007) and exhibited a V-shaped eyebrow, an open, downturned mouth and wide, open eyes. The stimulus had been rated as more highly threatening and arousing than all possible facial constructions of eyebrow shape (V-shaped and flat), mouth type (closed and open), eye type (wide and normal), and mouth curvature type (downturned and upturned) in previous research (Tipples, 2007). The neutral facial stimulus displayed a flat eyebrow, normal eye aperture, and a closed, expressionless mouth (see, Tipples, 2011).

The facial expressions measured 16 cm in height and 10.5 cm in width. Participants were seated 58 cm from the centre of the computer screen. When presented in the centre of the computer screen stimuli measured 16° of vertical visual angle. Stimuli were presented using 1 GHz Pentium computer connected to a 21.5-inch Dell ST2220 T computer monitor (1,920 × 1,080, 60 Hz). Participants sat at a comfortable distance from the computer screen but close enough to allow them to reach and touch the computer screen when necessary.

#### Design and procedure

Experiment 2 used a mixed 2 × 3 × 2 × 7 design with range (long and short) a between-subjects factor and action (push, pull, and no action), expression (angry and neutral), and duration (7) as three within-subjects factors.

Participants completed a modified version of the temporal bisection task in which participants were asked to judge whether stimuli are presented for a period of time closer to a previously learned *short* or *long* time duration. Participants were instructed to click the trigger button with their index finger using a Logitech Attack 3 computer joystick to indicate *short* or click a second button with their thumb to indicate *long*. The *short* duration in the long stimulus duration range was 400 ms while the *long duration* was 1,600 ms, compared with a *short* duration of 200 ms and a *long duration* of 800 ms in the short stimulus duration range.

The procedure for both the long and short stimulus duration range was identical and was based on the temporal bisection task used in Experiment 1. Participants were first taught to distinguish between the respective *short* and *long* time duration using a pink oval in a learning phase. For the first eight trials, the pink oval was presented in a fixed sequence (i.e., *short, long, short*, and *long*) followed by a further eight trials in which the pink oval randomly appeared for either the *short* or *long duration.* Therefore, during the learning phase, the pink oval was presented a total of 16 times, eight of which demonstrated the *short* time duration and eight demonstrating the *long duration.* During the learning phase, participants received feedback as to whether their response was correct; *YES* was presented in the middle of the screen in green ink if the participant correctly identified the stimulus as either *short* or *long* whereas *NO* appeared in red if their response was incorrect. Feedback was presented for 1,000 ms immediately after the participant responded.

In the testing phase, angry and neutral facial expressions were presented for either of the two previously learned durations (200/400 and 800/1,600 ms) or an intermediate duration (300/600, 400/800, 500/1,000, 600/1,200, and 700/1,400 ms). Participants were presented with an action instruction of either *pull, push*, or *no action*; the action instruction was presented for 1 s. After the action instruction, a facial expression displaying either an angry or neutral expression was presented for one of the stimulus durations. Following the presentation of the facial expression, a fixation cross was presented for three and a half seconds during which participants had to either *pull* or *push* the computer joystick or produce *no action*. Finally, participants were tasked with judging whether the presented facial stimulus was shown for a duration closer to either the *short* or *long* time duration which they had learnt in the learning phase; there was no time limit for participants to make this response. There was an intertrial interval of 500 ms throughout the experiment.

In total, each participant completed 504 trials derived from eight presentations of each facial expression across each time duration (7) and action preparation condition (3) separated into six blocks of 84 trials. Trials were randomly presented across the six blocks of trials.

### Results

**Figure 4.**
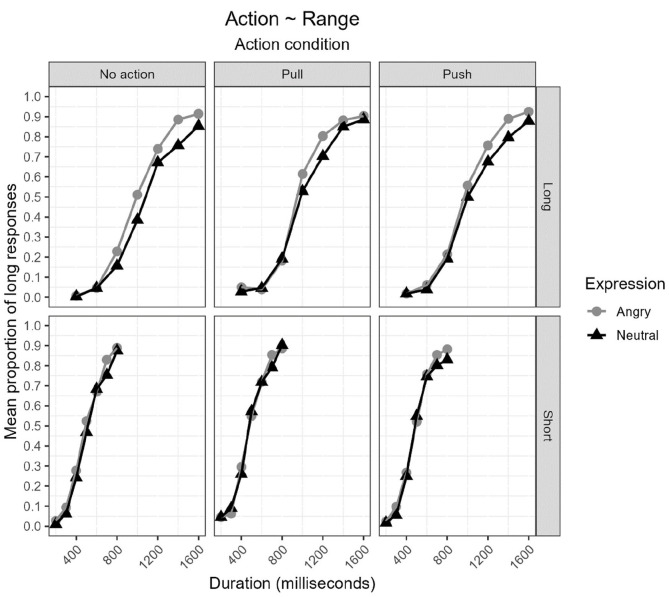
Proportion of long responses [*p*(long)] plotted against durations (ms) for the short-duration range group (bottom) and long-duration range group (top) for each combination of action condition (no action, pull, and push) and expression (angry and neutral).

Figure 4 displays the proportion of long responses [*p*(long)] plotted against durations (ms) for the short-duration range group (bottom) and long-duration range group (top) for each combination of action condition (no action, pull, and push) and expression (angry and neutral).

#### Model comparison

##### BPs

The four models with most support relative to the null are shown in Table 2 (top). Bayes factor analyses determined that the data were best represented by a model that included main effects of expression, (duration) range, action, and both the expression × range and expression × action interaction terms. The Bayes factor (BF10) was 6.91 × 10^216^ indicating decisive evidence in favour of this model when compared with the null model. Evidence favouring the top model was “anecdotal” (BF10 = 1.39) when compared with the second model as shown in Table 2. The second model included the additional action × range interaction term.

**Table 2.** Bayes factors relative to null (grand mean and intercept only) for BPs, WRs, JNDs, and reaction times (RTs) (for pulling and pushing the joystick) for Experiment 2. The four models with most support for each index (e.g., BPs) relative to the null are presented in rank order.

BP models	BF10
1. Expression + Action + Expression:Action + Range + Expression:Range + Expression:Subject + Subject	6.91 × 10^216^
2. Expression + Action + Expression:Action + Range + Expression:Range + Action:Range + Expression:Subject + Subject	5.05 × 10^216^
3. Expression + Action + Range + Expression:Range + Expression:Subject + Subject	2.65 × 10^216^
4. Expression + Action + Range + Expression:Range + Action:Range + Expression:Subject + Subject	1.61 × 10^216^
WR models	
1. Range + Subject	4.00 × 10^41^
2. Subject	2.10 × 10^41^
3. Expression + Range + Subject	4.37 × 10^40^
4. Action + Range + Subject	2.83 × 10^40^
JND models	
1. Range + Subject	1.00 × 10^59^
2. Expression + Range + Subject	5.00 × 10^58^
3. Expression + Range + Expression:Range + Subject	2.78 × 10^58^
4. Action + Range + Subject	3.54 × 10^57^
RTs	
1. Action + Range + Subject + Subject:Range	1.28 × 10^553^
2. Range + Subject	3.32 × 10^552^
3. Action + Range + Subject	1.68 × 10^552^
4. Action + Range + Action:Range + Subject:Action + Subject	1.09 × 10^552^

BP: Bisection point; JND: just noticeable difference; RTs: reaction times; WR: Weber ratio.

As shown in Figure 2 (right), the expression × range interaction showed that the effect of emotion was significantly smaller in magnitude for participants in the short-range condition compared with the long-range condition. Specifically, for the short duration, the BP was reached sooner for angry (*M* *=* 519) compared with neutral (*M* *=* 532) expressions, and this effect was medium to small in standardised units (Cohen’s *d*_z_ = −0.41, 95% HDI = [−0.80, −0.072]; 13 ms in magnitude; 95% HDI = [−25ms, −2 ms]). For the long-duration range condition, the BP was also reached sooner for angry (*M* *=* 1,034) compared with neutral (*M* *=* 1,070) expressions, and the effect was more than four times larger (63 ms in magnitude; 95% HDI = [−96, −32]) with standardised indices indicating a medium to large effect, Cohen’s *d*_z_ = −0.76, 95% HDI = [−1.22, −0.35]. In both cases, less than 1% of the HDI for the effect sizes fell within the ROPE (Cohen’s *d*_z_ _=_ −0.1 to 0.1).

The main effect of action condition showed that asking participants to prepare to act led to an overestimation effect with lower BPs for both the “push” condition compared with the “no-action” condition (Cohen’s *d*_z_ = 0.36, 95% HDI = [0.11, 0.62]; 20 ms in magnitude; 95% HDI = [6 ms, 34 ms]) and the “pull” condition compared with the “no-action” condition (Cohen’s *d*_z_ = 0.47, 95% HDI = [0.20, 0.76]; 32 ms in magnitude; 95% HDI = [15 ms, 48 ms]). Effect size differences between the push and pull conditions were very small with 24% of the difference falling within the ROPE (Cohen’s *d*_z_ = −0.19, 95% HDI = [−0.47, 0.06]; 12 ms in magnitude; 95% HDI = [−28 ms, 4 ms]).

The expression × action interaction showed that the instruction to act (push or pull) reduced the overestimation effect for angry compared with neutral expressions. Specifically, under conditions not to act (“Hold still”), there was a substantive leftward shift in the BP for angry versus neutral expressions (55 ms in magnitude; 95% HDI = [−77 ms, −34 ms]; Cohen’s *d*_z_ = −0.73, 95% HDI = [−1.08, −0.39]). The latter effect was more than halved in size when participants were instructed to either push (31 ms in magnitude; 95% HDI = [−59 ms, −5 ms]; Cohen’s *d*_z_ = −0.34, 95% HDI = [−0.62, −0.07]) or pull (31 ms in magnitude; 95% HDI = [−31 ms, 4 ms]; Cohen’s *d*_z_ = −0.13, 95% HDI = [−0.51, 0.06]) the joystick. For the pull condition specifically, the effect sizes were practically equivalent to zero—6.75% of the posterior fell within the ROPE (Cohen’s *d*_z_ = −0.13, 95% HDI = [−0.51, 0.06]).

##### WRs and JNDs

Following Experiment 1, for JNDs, Bayes factor showed that the best-fitting model included duration range as the sole factor (BF10 = 8.41 × 10^61^). JNDs were smaller for short-duration group (*M* = 108) compared with the long-duration range group (*M* = 184 ms), and the difference was large, Cohen’s *d*_z_ = −1.49, 95% HDI = [−2.17, −0.82]. As shown in Table 2 (middle), Bayes factors favoured the model that included the main effect of range with higher WRs (lower sensitivity) for the short range (*M* *=* .20) compared with the long-range condition (*M* *=* .17). This finding is supported by graphical superimposition (Figure 3, right) where the curve for plot of the proportion of long responses against the normalised duration is flatter (hence, WRs are higher) for the short versus the long-duration range. Overall, however, the difference in WRs was small with more than 5% of the standardised effect size posterior distribution falling with the ROPE (Cohen’s *d*_z_ = 0.46, 95% HDI = [−0.034, 0.969]). Moreover, evidence favouring best-fitting model versus the second model with range removed was anecdotal (BF10 = 1.90). In sum, the data appear to violate SET although the effect is small.

##### Superimposition

Following Experiment 1, a Bayesian multilevel logistic regression model was estimated as a further test for scalar invariance. The model was identical to that used in Experiment 1. Model results support the analyses of WRs namely the sign of the two-way interaction supported a relative decrease in the gradient of the slope for transformed duration for the short compared with the long duration, *b*-transformed duration × range (short) Log Odds *=* −0.80. The effect was small with 90% of the HDI excluding the value zero (−1.58, −0.001) rather than 95% of the HDI = [−1.74, 0.22]). The pd for the interaction term was 95.03%—we can be 95% certain the slope is negative.

##### RTs

RTs for pushing and pulling the joystick were also modelled. Trials on which participants moved the joystick in the wrong direction were first excluded. Then, the entire samples of RTs were screened for outliers using the method described by Cousineau and Chartier (2010). After removal of outliers, median RTs were analysed by calculating Bayes factors using the generalTestBF function described above. Models included all possible combinations of action type (pull and push), expression (angry and neutral), and range (short and long). The top four models are displayed in Table 2. None of the models included expression as a fixed effect. The top model included main effects for both action and range—participants were faster to pull (*M* = .54) than push (*M* = .55) and faster to respond in the long-duration (*M* = .40) compared with short-duration (*M* = .65) range.

### Discussion

The results differ from Experiment 1 and are in keeping with either widening of an attentional gate or speeding of a pacemaker (or both) for the effect of emotion on time estimation. Specifically, the effect of emotion on timing—operationalised as the difference in BPs between angry and neutral facial expressions—grew in magnitude from the short to long duration. Put differently, participants started to categorise angry faces as “long” sooner for angry compared with neutral faces, and this effect was larger in magnitude when the absolute durations were longer. Results also replicate previous reports of an overestimation effect due to preparation to act—BPs were reached sooner when participants were required to either prepare to pull or push a lever compared with the no-action condition. Finally, results also support a shared process account between action preparation and emotion—there was a reduction in the magnitude of the emotion effect in the action preparation conditions suggesting that preparing to act had already increased arousal prior to the onset of the face.

## General discussion

The novelty of the current research comes from examining the effects of emotion on timing performance using different duration ranges while concurrently testing for the effects of visual cues (Experiment 1) and action preparedness (Experiment 2). Results from Experiment 2 are consistent with either pacemaker speeding or widening of the attentional gate—the overestimation effect due to emotion grew in magnitude from the short- to the long-duration range. Also, for Experiment 2, emotion effects—conceptualised as shifts in the BP between angry and neutral expressions—were reduced in magnitude when participants were instructed to prepare to act. The latter finding suggests shared processing resources between action preparation and emotion and, more broadly, is in keeping with a central role for action in emotion states (Frijda, 2004).

A more difficult pattern to explain is the underestimation effect reported for angry (vs. neutral) faces reported for participants in the short-duration range condition in Experiment 1 (but not Experiment 2). Other studies have reported similar switches in the direction of effects due to emotion (Angrilli et al., 1997; Smith et al., 2011) including one study (Smith et al., 2011) where a reversal effect was reported for emotional images displayed for very short durations (100–300 ms). However, in this study, the underestimation effect for angry expressions was not replicated across studies. In Experiment 2, an overestimation effect of reduced magnitude was recorded for faces presented for short durations (200–800 ms). We do not have an explanation for the underestimation effect reported for angry faces displayed for short durations in Experiment 1 beyond the more general speculation that the effect may have been due to some specific aspect of either the faces (e.g., face sex) or the participants (e.g., individual differences in emotionality; Tipples, 2008).

Both visual cues and instructions to prepare to act distorted timing performance, and therefore, our tasks demonstrated sensitivity to the effects reported in past research. Both the effects of visual cues and preparation to act are consistent with a general increase in alertness—there was little support for either location-specific or action-specific effects. For example, both central and peripheral cues led to an overall overestimation effect relative to a no-cue condition. There was little support for timing starting sooner in the central cue condition compared with the peripheral cue as we might expect if a peripheral cue delayed the closing of an attentional control switch. Similarly, when instructed to prepare to act, overestimation was similar in magnitude across the push and pull conditions. Comparing the two manipulations, effect sizes were larger for visual cues (e.g., central vs. no-cue; Cohen’s *d*_z_ = −0.74, 95% HDI = [−1.07, −0.44]) compared with action preparation (e.g., push vs. no action; Cohen’s *d*_z_ = 0.36, 95% HDI = [0.11, 0.62]) perhaps illustrating a more central role for visual information in timing in this context.

The lack of differential effects for visual cues may be because peripheral and central visual cues differed in location along the horizontal plane only. When such cues possess depth—they are manipulated to either approach the viewer (“looming”) or recede from view—differential effects are found with looming and not receding cues producing temporal dilation effects (New & Scholl, 2009). Although the latter effects are differential (they occur for one cue and not the other), the authors found little support for spatial selectivity. Instead, the overall pattern supported the same general overestimation pattern reported here—a general alerting rather than a spatially graded effect.

Results are not fully consistent with SET (Church & Deluty, 1977; Gibbon, 1977; Gibbon et al., 1984)—they do not offer convincing support for scalar invariance for two reasons. First, despite apparently good *visual* superimposition, Bayes factor analyses of the WRs calculated for both experiments supported the inclusion of the variable “range” in the second of the top four models with evidential support (relative to the null model) for Experiment 1 and for the model with the highest Bayes factor for Experiment 2. Second, Bayesian multilevel logistic regression that included normalised durations indicated lower temporal sensitivity in the short-, compared the long-duration range condition even though the effect was relatively small in magnitude.

Separate research that recorded verbal estimates of temporal performance showed that presenting a flickering visual cue prior to (Wearden et al., 2017) or concurrently with a to-be-timed stimulus (Treisman & Brogan, 1992) leads to an overestimation effect. Moreover, the former study (Wearden et al., 2017) showed that the overestimation effect increased with the duration of the stimulus—a slope effect that is consistent with visual flicker increasing the rate of pulses emitted by the pacemaker proposed by SET. We also used a flickering visual cue presented in a central location but failed to record a slope effect. However, there are salient differences between our study and that of Wearden et al. that make a straightforward comparison difficult. First, we presented a very brief (200 ms) flickering cue in the study by Wearden et al. the total flicker period lasted for 6,000 ms. The brief cue duration used here is consistent with previous research that has used visual cues to probe attentional effects (Yantis & Jonides, 1990). Such briefly presented cues are thought to capture visual attention and simultaneously produce an increase in alertness (also an attentional effect). In other words, the short-duration cues used here likely target an attentional mechanism. Second, Wearden et al. used a verbal estimation task that may be more sensitive to slope effects. Future studies should address the above points manipulating visual flicker duration across task type (temporal bisection and verbal estimation) and duration range (short and long).

A further useful step in research will be to include measures and manipulations of emotional state. We do not know to what extent the stimuli used here created a state of arousal and, therefore, have adequate potency. Although multiplicative effects were found, a different set of results if either individual differences in emotional state had been measured and correlated with key timing effects or levels of arousal had been manipulated (e.g., Droit-Volet et al., 2011). Indeed, such effects have been found previous work (e.g., Angrilli et al., 1997; Droit-Volet et al., 2011; Pollatos et al., 2014) and highlight the need for further research comparing arousal levels and examining self-reported emotional state.

Overall, this study corroborates recent research that recorded support for the pacemaker speeding hypothesis for emotional images (Grommet et al., 2019). Emotion effects grew in magnitude from the short-duration to long-duration range. They go further by suggesting an action preparation account of the emotion effect. Whether visual attentional processes are capable modulating the effect of emotion remains to be established.

## Supplemental Material

sj-csv-2-qjp-10.1177_17470218231172856 – Supplemental material for Temporal distortion for angry faces: Testing visual attention and action preparation accounts Supplemental material, sj-csv-2-qjp-10.1177_17470218231172856 for Temporal distortion for angry faces: Testing visual attention and action preparation accounts by Jason Tipples, Michael Lupton and David George in Quarterly Journal of Experimental Psychology

sj-csv-3-qjp-10.1177_17470218231172856 – Supplemental material for Temporal distortion for angry faces: Testing visual attention and action preparation accounts Supplemental material, sj-csv-3-qjp-10.1177_17470218231172856 for Temporal distortion for angry faces: Testing visual attention and action preparation accounts by Jason Tipples, Michael Lupton and David George in Quarterly Journal of Experimental Psychology

sj-docx-1-qjp-10.1177_17470218231172856 – Supplemental material for Temporal distortion for angry faces: Testing visual attention and action preparation accounts Supplemental material, sj-docx-1-qjp-10.1177_17470218231172856 for Temporal distortion for angry faces: Testing visual attention and action preparation accounts by Jason Tipples, Michael Lupton and David George in Quarterly Journal of Experimental Psychology

## References

[bibr1-17470218231172856] Angrilli A. Cherubini P. Pavese A. Mantredini S. (1997). The influence of affective factors on time perception. Perception & Psychophysics, 59(6), 972–982. 9270369 10.3758/bf03205512

[bibr2-17470218231172856] Bar-Haim Y. Kerem A. Lamy D. Zakay D. (2010). When time slows down: The influence of threat on time perception in anxiety. Cognition and Emotion, 24, 255–263. 10.1080/02699930903387603

[bibr3-17470218231172856] Church R. M. Deluty M. Z. (1977). Bisection of temporal intervals. Journal of Experimental Psychology: Animal Behavior Processes, 3(3), 216–228. 10.1037/0097-7403.3.3.216 881613

[bibr4-17470218231172856] Cohen J. (1988). Statistical power analysis for the behavioral sciences (Headingley Library 300.15195 COH; 2nd ed.). Lawrence Erlbaum Associates.

[bibr5-17470218231172856] Cousineau D. Chartier S. (2010). Outliers detection and treatment: A review. International Journal of Psychological Research, 3(1), 58–67. 10.21500/20112084.844

[bibr6-17470218231172856] Droit-Volet S. Fayolle S. L. Gil S. (2011). Emotion and time perception: Effects of film-induced mood. Frontiers in Integrative Neuroscience, 5, Article 33. 10.3389/fnint.2011.00033 PMC3152725 21886610

[bibr7-17470218231172856] Droit-Volet S. Mermillod M. Cocenas-Silva R. Gil S. (2010). The effect of expectancy of a threatening event on time perception in human adults. Emotion, 10(6), 908–914. 10.1037/a0020258 21171761

[bibr8-17470218231172856] Fayolle S. Gil S. Droit-Volet S. (2015). Fear and time: Fear speeds up the internal clock. Behavioural Processes, 120, 135–140. 10.1016/j.beproc.2015.09.014 26440426

[bibr9-17470218231172856] Fernandez-Duque D. Posner M. I. (1997). Relating the mechanisms of orienting and alerting. Neuropsychologia, 35(4), 477–486. 10.1016/S0028-3932(96)00103-0 9106276

[bibr10-17470218231172856] Frijda N. H. (2004). Emotions and action. In Manstead A. S. R. Frijda N. Fischer A. (Eds.), Feelings and emotions: The Amsterdam symposium (pp. 158–173). Cambridge University Press. 10.1017/CBO9780511806582.010

[bibr11-17470218231172856] Gan T. Wang N. Zhang Z. Li H. Luo Y. (2009). Emotional influences on time perception: Evidence from event-related potentials. NeuroReport, 20(9), 839–843. 10.1097/WNR.0b013e32832be7dc 19407669

[bibr12-17470218231172856] Gibbon J. (1977). Scalar expectancy theory and Weber’s law in animal timing. Psychological Review, 84(3), 279–325. 10.1037/0033-295X.84.3.279

[bibr13-17470218231172856] Gibbon J. Church R. M. (1990). Representation of time. Cognition, 37(1–2), 23–54. 10.1016/0010-0277(90)90017-E 2269007

[bibr14-17470218231172856] Gibbon J. Church R. M. Meck W. H. (1984). Scalar timing in memory. Annals of the New York Academy of Sciences, 423, 52–77. 10.1111/j.1749-6632.1984.tb23417.x 6588812

[bibr15-17470218231172856] Gil S. Droit-Volet S. (2011). “Time flies in the presence of angry faces” . . . depending on the temporal task used! Acta Psychologica, 136, 354–362. 10.1016/j.actpsy.2010.12.010 21276583

[bibr16-17470218231172856] Gil S. Niedenthal P. M. Droit-Volet S. (2007). Anger and time perception in children. Emotion, 7, 219–225. 10.1037/1528-3542.7.1.219 17352578

[bibr17-17470218231172856] Goodrich B. Gabry J. Ali I. Brilleman S. (2020). rstanarm: Bayesian applied regression modeling via Stan. https://mc-stan.org/rstanarm

[bibr18-17470218231172856] Grommet E. K. Droit-Volet S. Gil S. Hemmes N. S. Baker A. H. Brown B. L. (2011). Time estimation of fear cues in human observers. Behavioural Processes, 86(1), 88–93. 10.1016/j.beproc.2010.10.003 20971168

[bibr19-17470218231172856] Grommet E. K. Hemmes N. S. Brown B. L. (2019). The role of clock and memory processes in the timing of fear cues by humans in the temporal bisection task. Behavioural Processes, 164, 217–229. 10.1016/j.beproc.2019.05.016 31102605

[bibr20-17470218231172856] Hagura N. Kanai R. Orgs G. Haggard P. (2012). Ready steady slow: Action preparation slows the subjective passage of time. Proceedings of the Royal Society B: Biological Sciences, 279(1746), 4399–4406. 10.1098/rspb.2012.1339 PMC3479796 22951740

[bibr21-17470218231172856] Iwasaki M. Tomita K. Noguchi Y. (2017). Non-uniform transformation of subjective time during action preparation. Cognition, 160, 51–61. 10.1016/j.cognition.2016.12.011 28049041

[bibr22-17470218231172856] Kruschke J. Meredith M. (2021). BEST: Bayesian Estimation Supersedes the t-Test. R package version 0.5.4. https://CRAN.Rproject.org/package=BEST.

[bibr23-17470218231172856] Kruschke J. K. (2018). Rejecting or accepting parameter values in Bayesian estimation. Advances in Methods and Practices in Psychological Science, 1(2), 270–280. 10.1177/2515245918771304

[bibr24-17470218231172856] Lee M. D. Wagenmakers E.-J. (2013). Bayesian cognitive modeling: A practical course. Cambridge University Press. 10.1017/CBO9781139087759

[bibr25-17470218231172856] Makowski D. Ben-Shachar M. S. Chen S. H. A. Lüdecke D. (2019). Indices of effect existence and significance in the Bayesian framework. Frontiers in Psychology, 10, Article 2767. 10.3389/fpsyg.2019.02767 PMC6914840 31920819

[bibr26-17470218231172856] Marsh A. A. Ambady N. Kleck R. E. (2005). The effects of fear and anger facial expressions on approach- and avoidance-related behaviors. Emotion, 5(1), 119–124. 10.1037/1528-3542.5.1.119 15755225

[bibr27-17470218231172856] Morey R. D. Rouder J. N. (2018). BayesFactor: Computation of Bayes factors for common designs. https://CRAN.R-project.org/package=BayesFactor

[bibr28-17470218231172856] New J. J. Scholl B. J. (2009). Subjective time dilation: Spatially local, object-based, or a global visual experience? Journal of Vision, 9(2), Article 4. 10.1167/9.2.4 19271914

[bibr29-17470218231172856] Pollatos O. Laubrock J. Wittmann M. (2014). Interoceptive focus shapes the experience of time. PLOS ONE, 9(1), Article e86934. 10.1371/journal.pone.0086934 PMC3906083 24489807

[bibr30-17470218231172856] Rotteveel M. Phaf R. H. (2004). Automatic affective evaluation does not automatically predispose for arm flexion and extension. Emotion, 4(2), 156–172. 10.1037/1528-3542.4.2.156 15222853

[bibr31-17470218231172856] Rouder J. N. Morey R. D. Speckman P. L. Province J. M. (2012). Default Bayes factors for ANOVA designs. Journal of Mathematical Psychology, 56(5), 356–374. 10.1016/j.jmp.2012.08.001

[bibr32-17470218231172856] Schneider W. Eschman A. Zuccolotto A. (2002). E-prime (Version 1.2). Psychology Software Tools.

[bibr33-17470218231172856] Smith S. D. McIver T. A. Di Nella M. S. J. Crease M. L. (2011). The effects of valence and arousal on the emotional modulation of time perception: Evidence for multiple stages of processing. Emotion, 11(6), 1305–1313. 10.1037/a0026145 22142208

[bibr34-17470218231172856] Tipples J. (2007). Wide eyes and an open mouth enhance facial threat. Cognition and Emotion, 21(3), 535–557.

[bibr35-17470218231172856] Tipples J. (2008). Negative emotionality influences the effects of emotion on time perception. Emotion, 8(1), 127–131. 10.1037/1528-3542.8.1.127 18266523

[bibr36-17470218231172856] Tipples J. (2011). When time stands still: Fear-specific modulation of temporal bias due to threat. Emotion, 11(1), 74–80. 10.1037/a0022015 21401227

[bibr37-17470218231172856] Tipples J. (2019). Increased temporal sensitivity for threat: A Bayesian generalized linear mixed modeling approach. Attention, Perception, & Psychophysics, 81(3), 707–715. 10.3758/s13414-018-01637-9 PMC6407721 30515645

[bibr38-17470218231172856] Tottenham N. Tanaka J. W. Leon A. C. McCarry T. Nurse M. Hare T. A. Marcus D. J. Westerlund A. Casey B. J. Nelson C. (2009). The NimStim set of facial expressions: Judgments from untrained research participants. Psychiatry Research, 168(3), 242–249. 10.1016/j.psychres.2008.05.006 19564050 PMC3474329

[bibr39-17470218231172856] Treisman M. Brogan D. (1992). Time perception and the internal clock: Effects of visual flicker on the temporal oscillator. European Journal of Cognitive Psychology, 4, 41–70. 10.1080/09541449208406242

[bibr40-17470218231172856] van Doorn J. Aust F. Haaf J. M. Stefan A. M. Wagenmakers E.-J . (2023). Bayes factors for mixed models. Computational Brain & Behavior, 6, 1–13. 10.1007/s42113-021-00113-2 PMC9981503 36879767

[bibr41-17470218231172856] Wearden J. H. Williams E. A. Jones L. A. (2017). What speeds up the internal clock? Effects of clicks and flicker on duration judgements and reaction time. Quarterly Journal of Experimental Psychology, 70(3), 488–503. 10.1080/17470218.2015.1135971 26811017

[bibr42-17470218231172856] Yantis S. Jonides J. (1990). Abrupt visual onsets and selective attention: Voluntary versus automatic allocation. Journal of Experimental Psychology: Human Perception and Performance, 16(1), 121–134. 10.1037/0096-1523.16.1.121 2137514

[bibr43-17470218231172856] Zakay D. Block R. A. (1996). The role of attention in time estimation processes. In Pastor M. A. Artieda J. (Eds.), Advances in psychology (Vol. 115, pp. 143–164). North-Holland. 10.1016/S0166-4115(96)80057-4

