# Supplementary material for: Temporal distortion for angry faces: Testing visual attention and action preparation accounts
Source: Q J Exp Psychol (Hove). 2023 May 30;77(9):1800–12. doi: 10.1177/17470218231172856 (PMC11373163; doi:10.1177/17470218231172856)
Supplement: sj-docx-1-qjp-10.1177_17470218231172856 – Supplemental material for Temporal distortion for angry faces: Testing visual attention and action preparation accounts [file sj-docx-1-qjp-10.1177_17470218231172856.docx]

# Supplementary Material for “Temporal Distortion for Angry Faces; Testing Visual Attention and Action Preparation Accounts”

**Jason Tipples^1,^*, Michael Lupton^2^ and David George^2^**

^1^ Psychology Group, Leeds Beckett University, Leeds, UK

^2^Department of Psychology, University of Hull, Hull, UK

Face Stimuli Used in Experiment 2

| 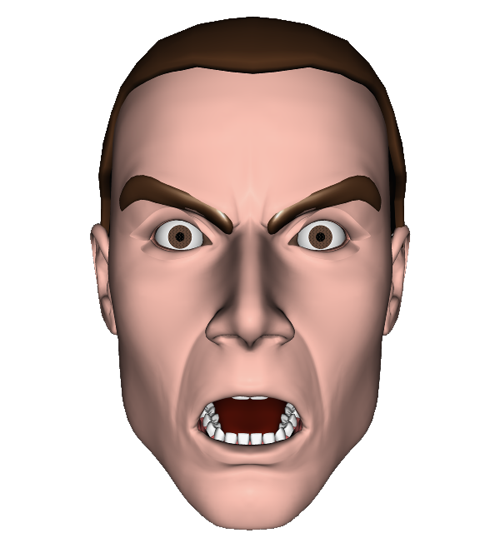 | 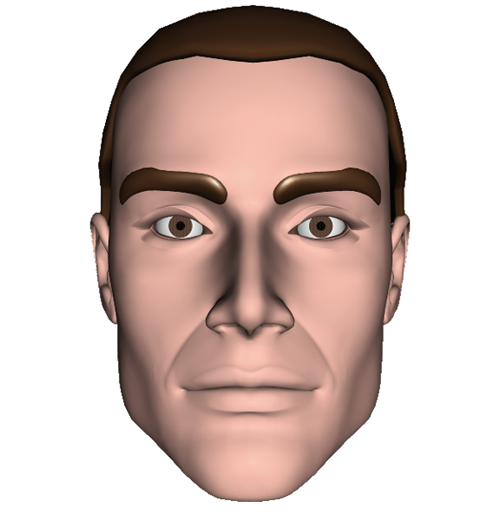 |
| --- | --- |
| Angry | Neutral |
